# Incidence of Lyme Carditis and Lyme Carditis as a Cause of Pacemaker Implantation: A Nationwide Registry-Based Case-Control Study

**DOI:** 10.1093/ofid/ofad656

**Published:** 2023-12-21

**Authors:** Sanna Avellan, Kirsten Mehlig, Josefina Robertson, Daniel Bremell

**Affiliations:** Department of Infectious Diseases, Region Västra Götaland, Sahlgrenska University Hospital, Gothenburg, Sweden; Department of Infectious Diseases, Institute of Biomedicine, Sahlgrenska Academy, University of Gothenburg, Gothenburg, Sweden; School of Public Health and Community Medicine, Institute of Medicine, University of Gothenburg, Gothenburg, Sweden; Department of Infectious Diseases, Region Västra Götaland, Sahlgrenska University Hospital, Gothenburg, Sweden; Department of Infectious Diseases, Institute of Biomedicine, Sahlgrenska Academy, University of Gothenburg, Gothenburg, Sweden; Department of Infectious Diseases, Region Västra Götaland, Sahlgrenska University Hospital, Gothenburg, Sweden; Department of Infectious Diseases, Institute of Biomedicine, Sahlgrenska Academy, University of Gothenburg, Gothenburg, Sweden

**Keywords:** *Borrelia burgdorferi*, Lyme borreliosis, Lyme carditis, Lyme disease

## Abstract

**Background:**

Lyme borreliosis (LB) of the heart is called Lyme carditis (LC), which often manifests with high-grade atrioventricular block (AVB) requiring pacemaker implantation. LC is treated with antibiotics, and most patients recover fully after treatment. The overall incidence of LC, and of LC as a cause of pacemaker implantation, has not previously been systematically studied.

**Methods:**

This was a case-control study based on data from Swedish national registers. The study was divided into two parts; part 1 including all patients diagnosed with AVB between 2001 and 2018, and part 2 including all patients who had received a pacemaker due to AVB between 2010 and 2018. Patients diagnosed with LB 90 days before and 180 days after the AVB diagnosis were identified among the patients and compared to matched control groups generated from the general population.

**Results:**

Of 81 063 patients with AVB, 102 were diagnosed with LB. In the control group, 27 were diagnosed with LB. The yearly incidence of LC was 0.056 per 100 000 adults and year. Of 25 241 patients who had received a pacemaker for AVB, 31 were diagnosed with LB. In the control group, 8 were diagnosed with LB. The yearly incidence of LC as a cause of pacemaker implantation was 0.033 per 100 000 adults and year. The estimated risk for patients with LC to receive a permanent pacemaker was 59%.

**Conclusions:**

LC is a rare cause of AVB. Nevertheless, more than half of patients with LC receive a permanent pacemaker for a condition that is easily cured with antibiotics.

## Teaser

In this nationwide registry–based study, we present data on the incidence of Lyme carditis, Lyme carditis as a cause of pacemaker implantation, and the risk for patients with Lyme carditis to receive a permanent pacemaker.

Lyme carditis (LC) is one of the manifestations of Lyme borreliosis (LB), the infectious disease caused by spirochetes of the *Borrelia burgdorferi* sensu lato (*Bb*) species. LC usually presents with atrioventricular conduction block (AVB), and the diagnosis is based on the cardiac manifestation in combination with a positive *Bb* serology [1]. LC often causes high-grade AVB (defined as second-degree AVB Mobitz type 2, or third-degree AVB), which is an indication for pacemaker implantation [2]. After treatment with antibiotics, the prognosis of LC AVB is good, with the majority of patients recovering fully within weeks after treatment. Implantation of a permanent pacemaker is therefore not recommended for patients with LC, even with high-grade AVB [1]. From previous epidemiological studies, the incidence of LC in Europe has been estimated at 0.3%–4% of patients with LB [3]. To date, there are no studies that have investigated the nationwide incidence of LC in patients with cardiac symptoms suggestive of LC. The aim of the present study was to estimate the incidence of LC and of LC as a cause of permanent pacemaker implantation in Sweden, a country highly endemic for LB [4].

## METHODS

In Sweden, all hospitalizations and hospital outpatient visits are recorded in the National Patient Register with diagnoses registered according to the *International Classification of Diseases* (*ICD*). All inhabitants in Sweden have a unique personal identification number, and it is therefore possible to combine data from different sources. In this case-control study, we used linked nationwide data from Swedish registers between 2001 and 2018.

The following data sources were used: the National Patient Register, which captures data on diagnoses from all hospital admissions, emergency departments, and specialized outpatient clinics in Sweden; Statistics Sweden, the national statistics agency; and the Swedish Implantable Cardioverter Defibrillator and Pacemaker Registry, which captures data from all pacemaker implanting clinics in Sweden.

### Cases and Controls

#### Part 1: Incidence of LC in Sweden

Part 1 of the study included all adults (aged >18 years) who had been diagnosed with AVB of any degree, either as hospital inpatients or at specialized outpatient clinics, between 2001 and 2018. In this group, we identified patients diagnosed with LB within a time period of 90 days before and 180 days after the AVB diagnosis. If a patient had received the AVB diagnosis more than once between 2001 and 2018, only the first episode was included. A control group, matched 1:1 according to age, sex, and city of residence, and who had not been diagnosed with AVB during the same time period, was generated from the general population. In the control group, all cases of LB during the specific time period were identified.

#### Part 2: Incidence of Pacemaker due to LC in Sweden

Part 2 of the study included all adults (aged >18 years) who had had a pacemaker implanted for AVB between 2010 and 2018. In this group, we identified patients diagnosed with LB within a time period of 90 days before and 180 days after the AVB diagnosis. A control group, who had not received a pacemaker for AVB during the same time period, was generated as above.

### Diagnostic Codes

The following *ICD, Tenth Revision* diagnostic codes were used: Lyme borreliosis (A69.2), first-degree AVB (I44.0), second-degree AVB Mobitz type I/Wenckebach (I44.1A), second-degree AVB Mobitz type II (I44.1B), third-degree AVB (I44.2), and AVB not otherwise specified (I44.3).

### Statistical Analysis

The total incidence of LB diagnoses was estimated in the matched control groups, that is, the baseline rate of LB, and compared to data from National Patient Register on the average incidence for LB among adults between 2001 and 2018 (2010–2018 in part 2). The number of cases of LC was estimated as the difference in number of LB cases among patients with AVB (LB_p_) and matched controls without AVB (LB_c_), that is, LC = LB_p_ – LB_c_. The size of the total adult population (TA) was obtained from Statistics Sweden, and estimated as 7.5 × 10^6^ between 2001 and 2018 (TA_pop1_) and 7.8 × 10^6^ between 2010 and 2018 (TA_pop2_).

The incidence of LC in the adult population was given by the calculated number of cases (LB_p_ – LB_c_) divided by the total person-time, given by 18 years multiplied with the average size of the adult Swedish population during these years, that is, (LB_p_ – LB_c_) / (18 years × TA_pop_). Because the incidence of LC was estimated as the difference between number of LB among patients with AVB (LB_p_) and the number of LB in matched controls (LB_c_), with both independent of each other, the variance of LC diagnoses was given by LB_p_ + LB_c_. Confidence intervals (CIs) for LC incidence were given by ± 1.96 $\sqrt{LB_{p}+LB_{c}}$ / (18 years × TA_pop_). The incidence of LC among patients who had received a pacemaker for AVB was estimated using the same method.

## RESULTS

### Part 1: Incidence of LC in Sweden

**Figure 1.**
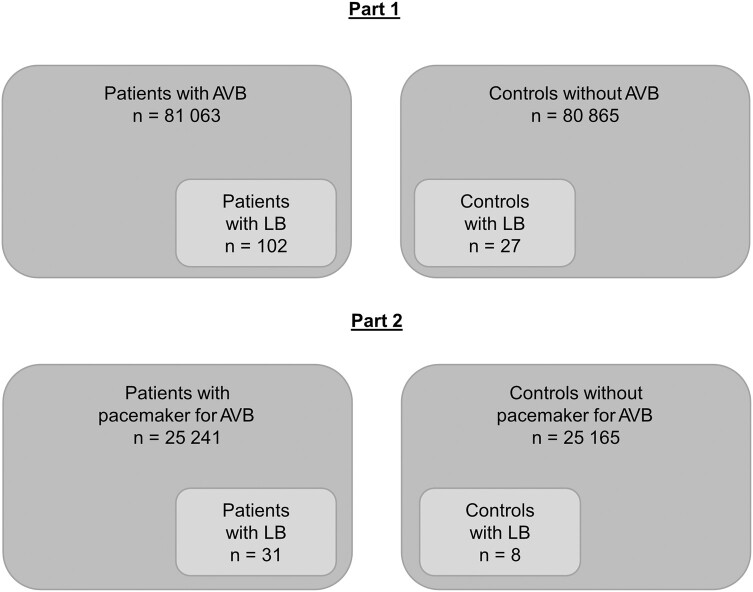
Schematic illustration of patients and controls. Abbreviations: AVB, atrioventricular block; LB, Lyme borreliosis.

**Figure 2.**
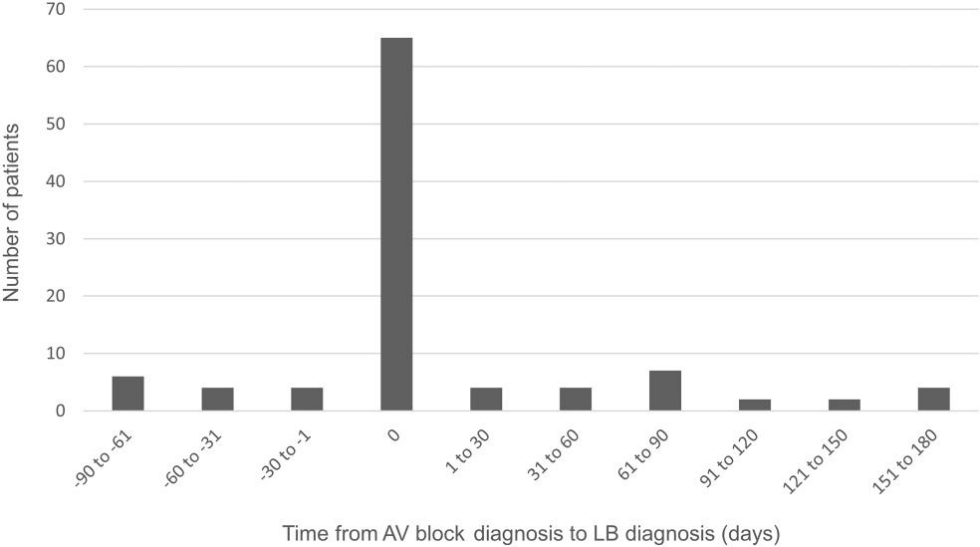
Time (days) from atrioventricular block (AVB) diagnosis to Lyme borreliosis (LB) diagnosis for patients in part 1, with both AVB and LB (n = 102).

**Figure 3.**
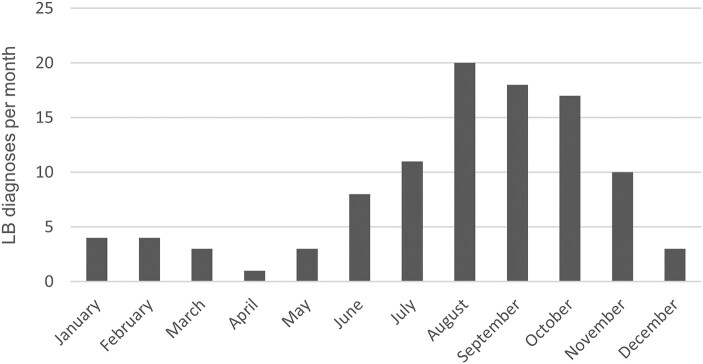
Number of Lyme borreliosis (LB) diagnoses per month for patients in part 1 with both atrioventricular block and LB (n = 102).

Between 2001 and 2018, 81 063 patients were diagnosed with AVB of any degree. Of these, 43 694 (54%) had high-grade AVB. One hundred two patients were diagnosed with LB within 90 days before and 180 days after the AVB diagnosis, and 73 of them (72%) had high-grade AVB. The median age of these 102 patients was 60 years (range, 18–92 years). Twenty-eight were female and 74 were male (Table 1, Figure 1). Sixty-five of the 102 patients with LB (64%) were diagnosed with AVB and LB on the same date, whereas 37 patients received the LB diagnosis either before or after the AVB diagnosis (Figure 2). The time of year when the AVB patients received their LB diagnosis varied throughout the year with a peak from August to October (Figure 3). For 198 cases with AVB, a matched control could not be found. Hence, the matched control group comprised 80 865 persons without AVB, of which 27 patients were diagnosed with LB. This corresponds to a yearly incidence of 33 (95% CI, 21–46) cases of LB per 100 000 adults and year. From the National Patient Register, the average incidence of LB among adults aged 20 years or older in Sweden between 2001 and 2018 was 29 per 100 000 inhabitants and year.

**Table 1.** Patient Characteristics

Characteristic	Value
Part 1: Incidence of LC in Sweden, 2001–2018 (18 y)	
Patients with AVB	81 063
LB	102
Age, y, median (range)	60 (18–92)
Sex, male/female	74/28
High-grade AVB, No. (%)	73 (72%)
Matched controls	80 865
LB	27
Incidence of LB, per 100 000 and year (95% CI)^a^	33 (21–46)^b^
Incidence of LC, per 100 000 and year (95% CI)^a^	0.056 (.039–.072)^c^
Part 2: Incidence of pacemaker due to LC in Sweden 2010–2018 (9 y)	
Patients with pacemaker due to AVB	25 241
LB	31
Age, y, median (range)	74 (20–92)
Sex, male/female	23/8
High-grade AVB, No. (%)	28 (90%)
Matched controls	25 165
LB	8
Incidence of LB, per 100 000 and year (95% CI)^a^	32 (10–54)^d^
Incidence of LC with pacemaker, per 100 000 and year (95% CI)^a^	0.033 (.015–.050)^e^
Parts 1 + 2: Fraction of LC patients with pacemaker	0.033/0.056 × 100% = 59%

Data are presented as No. unless otherwise indicated.

Abbreviations: AVB, atrioventricular block; CI, confidence interval; LB, Lyme borreliosis; LC, Lyme carditis.

^a^Incidence estimates with 95% CI = point estimate ± 1.96 SE, Standard Error.

^b^SE = $\sqrt{27}$/80 865.

^c^SE = $\sqrt{102+27}$ /18/7.5 × 10^6^.

^d^SE = $\sqrt{8}$ /25 165.

^e^SE = $\sqrt{31+8\;}$ /18/7.8 × 10^6^.

The number of cases of LC during 2001–2018 was calculated as the difference in number of cases of LB between the patients (n = 102) and the controls (n = 27), that is, 75 cases. The incidence of LC in the entire adult population, corrected for unrelated *Borrelia* infection, was 0.056 (95% CI, .039–.072) cases per 100 000 adults and year.

### Part 2: Incidence of Pacemaker due to LC

Between 2010 and 2018, 25 241 patients had a pacemaker implanted on AVB indication. Out of these, 31 patients had an LB diagnosis within 90 days before and 180 days after the AVB diagnosis (Table 1, Figure 1). For 76 cases, a matched control could not be found, and therefore, there were 25 165 persons in the matched control group. In the control group with neither pacemaker nor AVB, there were 8 patients with LB, corresponding to an estimated incidence of LB of 32 cases per 100 000 adults and year (95% CI, 10–54). The number of cases of LC as the cause of pacemaker implantation between 2010 and 2018 was calculated as the difference in number of cases of LB between the patients (n = 31) and the controls (n = 8), and equal to 23 cases. The incidence of pacemaker implantation due to LC in the entire adult population, corrected for unrelated *Borrelia* infection, was 0.033 cases per 100 000 adults and year (95% CI, .015–.050).

Combining incidence rates from both parts of the study, the estimated fraction of LC patients who received a permanent pacemaker was given by 0.033 / 0.056 = 0.59 (ie, 59%) (Table 1).

## DISCUSSION

In this register-based case-control study, we investigated the incidences of LC and LC-associated pacemaker implantation in Sweden. We found the LC incidence to be 0.056 cases per 100 000 adults and year, and the incidence of a permanent pacemaker implantation due to LC was 0.033 cases per 100 000 adults and year. Combining the data, the estimated risk for patients with LC to receive a permanent pacemaker was 59%, indicating a considerable percentage of pacemaker implantations that could have been avoided.

The most common manifestation of LC is AVB, being the presenting symptom in approximately 90% of cases with LC [5]. We investigated the incidence of LB in patients with AVB compared to a matched control group without AVB, based on the hypothesis that the percentage of patients with LB would be higher in the AVB group than in the non-AVB group, and that the difference in incidence would be a good estimate of the incidence of LC. Control groups were matched according to age, sex, and city of residence because the occurrence of ticks transmitting the *Borrelia* spirochetes varies between different regions of the country and because earlier studies have shown that both higher age and male sex increase the risk of LC and of AVB [6–8].

The wide time interval (270 days) to search for LB diagnosis was chosen for two reasons: The maximum incubation time for LC has been described to be up to 7 months, and since LC is a rare diagnosis, there is a risk of both patient's and doctor's delay [9]. With a wide timespan, we would be able to detect patients who had received the LB diagnosis at one clinic and the AVB diagnosis at another clinic, at a different time point. However, 65 of 102 patients in the AVB group were diagnosed with both AVB and LB on the same date. The remaining 37 cases were evenly distributed within the time span, and thus, most of the supposed LC cases would have been found even without the use of the 90 plus 180-day interval (Figure 2).

The patients with AVB and LB showed the same seasonal variance in LB diagnosis as is seen for other disseminated manifestations of LB, such as neuroborreliosis (Figure 3) [10]. This indicates that the incubation period for LC does not differ significantly from other manifestations of LB where most patients experience symptoms within 1 month after the tick bite [11]. Among patients with both AVB and LB, the male-to-female sex ratio in our study (2.6:1) is similar to that previously reported for LC (3:1) [12]. This sex difference sets LC apart from other *Borrelia* manifestations, such as erythema migrans, that are more common among women [13].

LB differs between Europe and the United States (US). In the US, *Bb* sensu stricto is the genospecies responsible for almost all human cases of LB [11]. It has been speculated that this genospecies is more cardiotropic than the *Bb* genospecies more commonly seen in Europe and that this explains the observed difference in LC incidence between Europe and the US [14]. The difference complicates comparisons between US and European LC epidemiological studies, but since the distribution of *Bb* genospecies is relatively similar throughout Europe, the results of the present study should be generalizable to other European countries [15]. The only previous study that has examined the incidence of LC in Sweden found that 0.5% of all included patients with LB had LC [16]. As LB is not a notifiable disease in Sweden, the national incidence of LB is not known. The numbers described above from the National Patient Register (29 cases per 100 000 inhabitants and year) do not include data from primary care, where many cases of erythema migrans are diagnosed. A recent nationwide Swedish study on the incidence of Lyme neuroborreliosis calculated the overall LB incidence in Sweden to 39 cases per 100 000 inhabitants and year [4]. Using these numbers and the LC incidence from this present study (0.056 cases per 100 000 inhabitants and year), it can be estimated that 0.1% of Swedish LB patients have LC. It is usually stated that 0.3%–4% of European LB patients have LC [14]. These numbers are, however, based on small studies that are >30 years old [17]. It can be argued that the lower figure presented in the current study is more accurate as it is based on nationwide data and as the study was specifically designed to investigate the incidence of LC.

Almost all LC patients recover from the AVB after antibiotic treatment [18]. A minority of patients require a temporary pacemaker but due to the excellent prognosis, implantation of a permanent pacemaker is almost never needed [19]. The fact that an estimated 59% of LC patients in this present study received a permanent pacemaker is therefore a cause for concern. The reason could be that LC as a cause of AVB is not considered before the patient has received the pacemaker, or that the responsible physician is unaware of the excellent prognosis after antibiotic treatment. Either way, awareness of LC as a cause of AVB needs to be raised among cardiologists.

This study has some limitations. First, our data are based only on hospital inpatients and patients attending specialized outpatient clinics. LB and lower degree AVB diagnoses from primary healthcare are not registered in the National Patient Register and the number of cases has therefore very likely been underestimated. However, this was the case in both the study groups and control groups and should not have affected the comparison between groups. Also, LC mostly manifests with high-degree AVB, requiring hospital inpatient care and monitoring [12]. Second, there is a potential risk that some cases of LC were missed as we only included patients with AVB, and LC may cause other cardiac symptoms such as atrioventricular fibrillation, pericarditis, myocarditis, or (in rare cases) endocarditis [14]. LC without AVB is, however, rare—according to previous studies, approximately 10% of all LC cases [18, 19]. Third, as the study was based on registered diagnoses, it would not capture cases of LC where no LB diagnosis was registered. As *Borrelia* serology testing is recommended in the diagnostic algorithms for patients with AVB of unknown cause, the number of missed cases should, however, be small.

## CONCLUSIONS

Our findings show that Lyme carditis is a rare condition, with an incidence of 0.056 cases per 100 000 adults in Sweden, a country that is highly endemic for Lyme borreliosis. The study also shows that there is a high risk for patients with LC to receive a permanent pacemaker. As the condition is easily treated with antibiotics and fully reversible in most cases, better knowledge of the condition can hopefully lead to earlier diagnosis and treatment, and thereby that fewer patients will unnecessarily undergo pacemaker implantation.

## Notes

***Author contributions.*** Conceptualization and funding acquisition: D. B. Methodology: All authors. Investigation: S. A. and D. B. Analysis: S. A., K. M., and D. B. Writing–original draft: S. A. and D. B. Writing–review and editing: All authors.

***Patient consent.*** All data were anonymized prior to analysis, and a written consent from the participants was not required. The study design conforms to registry-based study standards and was approved by the Swedish Ethical Review Authority (Dnr 2019-05324).

***Financial support*.** This study was supported by grants from the Swedish State under the ALF agreement between the Swedish government and the county councils (grant numbers ALF-70150 and ALF-73490 to D. B. and S. A.; ALF- 942707 to J. R.).
